# Co-inhibitory Receptor Signaling in T-Cell-Mediated Autoimmune Glomerulonephritis

**DOI:** 10.3389/fmed.2020.584382

**Published:** 2020-11-04

**Authors:** Kei Nagai

**Affiliations:** Department of Nephrology, Faculty of Medicine, University of Tsukuba, Tsukuba, Japan

**Keywords:** glomerulonephritis, therapeutics, ITIM, PD-1, CTLA4, renal vasculitis

## Abstract

Autoimmune glomerulonephritis occurs as a consequence of autoantibodies and T-cell effector functions that target autoantigens. Co-signaling through cell surface receptors profoundly influences the optimal activation of T cells. The scope of this review is signaling mechanisms and the functional roles of representative T-cell co-inhibitory receptors in the regulation of autoimmune glomerulonephritis, along with current therapeutic challenges mainly on preclinical trials. Co-inhibitory receptors utilize both shared and unique signaling pathway, suggesting specialized functions that provide the rationale behind therapies for autoimmune glomerulonephritis by targeting these inhibitory receptors. These receptors largely suppress Th1 immunity, modify Th17 and Th2 immune response, and enhance Treg function. Anti-cytotoxic T-lymphocyte-associated protein 4 (CTLA4) immunoglobulin (Ig), which is able to block both activating CD28 and inhibitory CTLA4 signaling, has been shown in preclinical and clinical investigations to have effects on glomerular disease. Other inhibitory receptors for treating glomerulonephritis have not been clinically tested, and efficacy of manipulating these pathways requires further preclinical investigation. While immune checkpoint inhibition using anti-CTLA4 antibodies and anti-programmed cell death 1 (PD-1)/PD-L1 antibodies has been approved for the treatment of several cancers, blockade of CTLA4 and PD-1/PD-L1 is associated with adverse effects that resemble autoimmune disorders, including systemic vasculitis. A renal autoimmune vasculitis model features an initial Th17 dominancy followed later by a Th1-dominant outcome and Treg cells that attenuate autoreactive T-cell function. Toward the development of effective therapies for T-cell-mediated autoimmune glomerulonephritis, it would be preferable to pay attention to the impact of the inhibitory pathways in immunological renal disease settings.

## Introduction

T cells are key effectors of the adaptive immune response, playing important roles in the elimination of pathogens and in the development of autoimmune disease. Autoimmune glomerulonephritis occurs as a consequence of autoantibodies and T-cell effector functions that target either antigens intrinsic to the glomeruli [for example, as occurs in anti-glomerular basement membrane (GBM) nephropathy] or non-specific antibodies that become trapped and accumulate in the glomeruli [for example, as occurs in immunoglobulin (Ig) A nephropathy and anti-neutrophil cytoplasmic autoantibody (ANCA)-associated glomerulonephritis] (1, 2). In this regard, peripheral regulation of T-cell responses is crucial to preventing inappropriate responses to self-antigens leading to autoimmune glomerulonephritis (1).

The optimal activation of T cells is profoundly influenced by co-signaling through cell surface receptors (3). The common feature that identifies receptors as members of the inhibitory class is their ability to attenuate activation signals initiated by other receptors that are often members of the immunoreceptor tyrosine-based activation motif (ITAM) class (4). Loss of inhibitory signaling is often associated with autoreactivity and unchecked inflammatory responses, illustrating the essential role that this system plays in immune regulation (4, 5). Though the human genome is estimated to encode over 300 immunoreceptor tyrosine-based inhibitory motif (ITIM)-containing molecules, of which only a minority has been characterized (6), most reviews discussing the co-receptor signaling pathway as a potential target in autoimmunity have focused on blockade of co-stimulatory receptor signaling (7, 8). The scope of this review is signaling mechanisms and the functional roles of some representative T-cell co-inhibitory receptors in the regulation of autoimmune glomerulonephritis, along with current therapeutic challenges.

## Classical and Non-classical Inhibitory Signaling in T-cell Receptor Pathway

### TCR Signaling Pathway and Co-signaling

**Figure 1:**
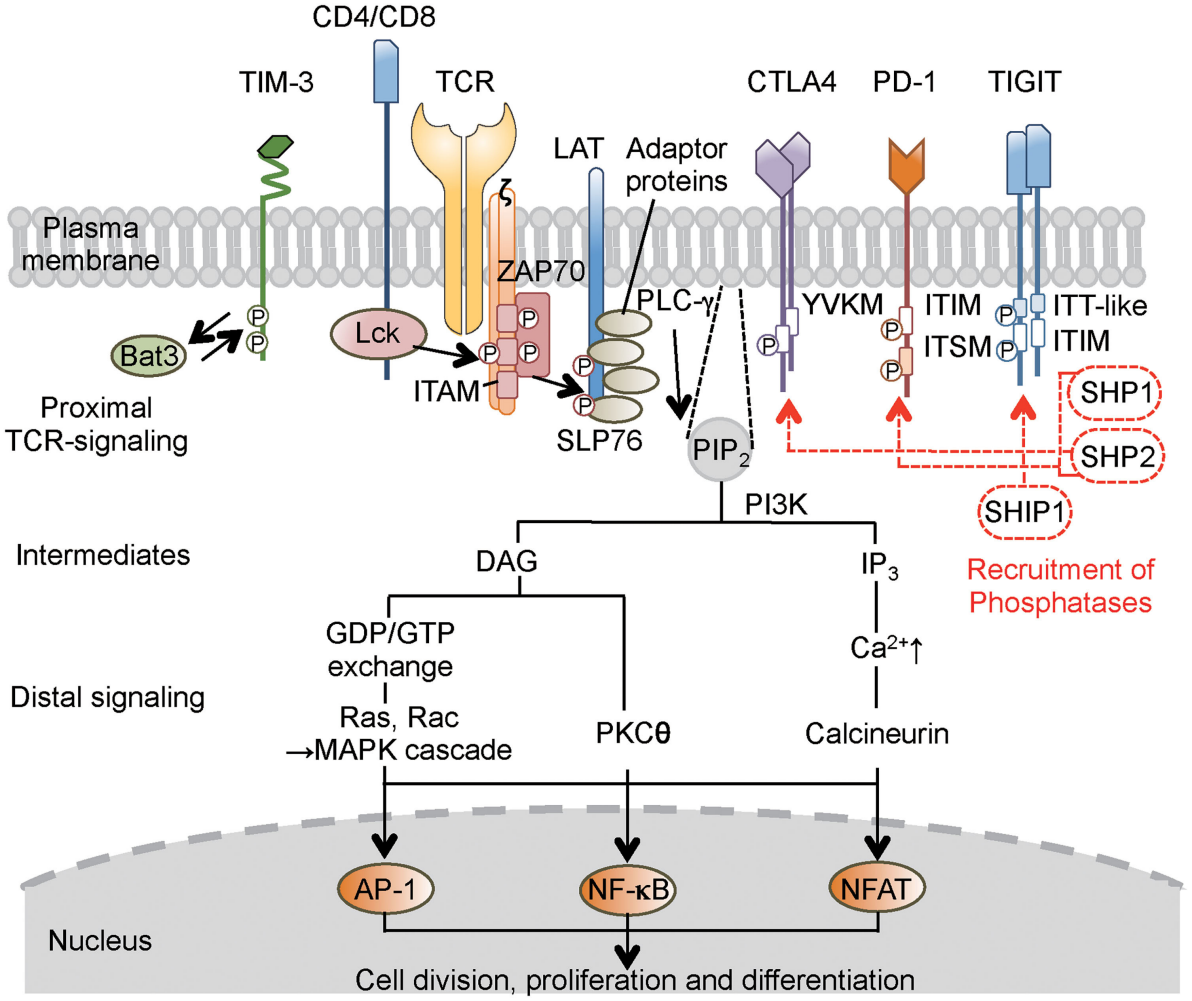
Concise outline of the intracellular signaling events during T-cell activation and roles of co-inhibitory receptors. Binding of the TCR and co-receptors to peptide–MHC (major histocompatibility complex) complexes on the antigen-presenting cells (APCs) initiates proximal signaling events, which result in phosphorylation of the ζ chain, binding and activation of ZAP-70, phosphorylation of LAT and adaptor proteins, production of biochemical intermediates, and activation of distal signaling cascades. MAPK, PKC, and calcineurin are enzymes that activate transcription factors, thereby stimulating the expression of various genes involved in T-cell response. Most inhibitory receptors have an inhibitory motif, represented by ITIM, in their cytoplasmic tails. Ligand binding to these receptors results in the recruitment of phosphatases (SHP-1, SHP-2, or SHIP1), which alter proximal and distal TCR signals. These consequently transmit activating signal (black solid line arrow) and inhibitory signal (red broken line and arrow) in T cells. In the case of TIM-3, which does not have a classical signaling motif, Bat-3 (HLA-B associated transcript 3) binds to the TIM-3 tail and blocks binding of another adaptor molecule under steady state. Ligand binding triggers the dissociation of Bat-3 from the cytoplasmic tail of TIM-3, thus allowing another adaptor molecule to bind and promote the inhibitory function of TIM-3. CTLA4, cytotoxic T-lymphocyte-associated protein 4; PD-1, programmed cell death 1; TIM-3, T-cell immunoglobulin 3; TIGIT, T-cell immunoglobulin and ITIM domain; LAT, linker for activation of T cells; TCR, T-cell receptor; ZAP-70, **ζ**-associated protein of 70 kD; SLP-76, SH2-binding leukocyte phosphoprotein of 76 kD; ITIM, immunoreceptor tyrosine-based inhibitory motif; ITSM, immunoreceptor tyrosine-based switch motif; ITT, immunoglobulin tail tyrosine; SHP-1,-2, Src-homology-2 domain-containing protein tyrosine phosphatase 1, 2; SHIP1, SH2 domain-containing inositol-5-phosphatase 1; PIP_2_, phosphatidylinositol biphosphate; PI3K, phosphatidylinositol-3 kinase; DAG, diacylglycerol; IP_3_, phosphatidylinositol triphosphate; GDP/GTP, guanosine diphosphate/triphosphate; MAPK, mitogen-activated protein kinase; PLC, phospholipase C; PKC, protein kinase C; AP-1, activation protein 1; NF-κB, nuclear factor-κB; NFAT, nuclear factor of activated T cells.

The primary signal for conventional T cells is mediated through T-cell receptor (TCR) engagement. TCRs recognize small antigenic peptides presented in the groove of the self-major histocompatibility complex (MHC) (9). As a result of this recognition, TCR complexes aggregate on T-cell surfaces to form stable contacts, resulting in the formation of immunological synapses on antigen-presenting cells (APCs) (9). This aggregation evokes intracellular signaling that involves the activation of Src protein tyrosine kinase, leading to the phosphorylation of CD3- and ζ chain-localized ITAM. Subsequently, the **ζ**-associated protein of 70 kD (ZAP-70) is recruited, resulting in a series of downstream phosphorylation events (10) (Figure 1). Another kinase pathway in T cells involves the activation of phosphatidylinositol-3 kinase (PI3K), which phosphorylates a specific membrane-associated inositol lipid. This enzyme is recruited to the TCR complex and generates phosphatidylinositol triphosphate (PIP_3_) and diacylglycerol (DAG) from membrane phosphatidylinositol biphosphate (PIP_2_). PIP_3_ activates signaling enzymes such as PLCγ (phospholipase Cγ) and PKCθ (protein kinase Cθ). However, the primary signal itself does not decide the fate of the immune response (11). Instead, co-stimulatory and co-inhibitory receptors on T cells direct the function and fate. These co-signaling receptors often co-localize with TCR molecules, such that the co-signaling receptors synergize with TCR signaling to promote or inhibit T-cell activation and function (11, 12).

### ITIM, ITSM, ITT, and Other Mechanisms

Intracellular protein–protein interaction during cell signaling and activities of cellular enzymes are often regulated by phosphorylation of tyrosine residues. For countering action of phosphorylation by tyrosine kinase, protein tyrosine phosphatases are enzymes that remove phosphate moieties from tyrosine residues to limit and terminate cellular responses that are no longer required (4, 13). One family of immune inhibitory receptors is defined by the presence of a consensus amino acid sequence, the ITIM motif, in the cytoplasmic domain of the proteins (13). The six-amino acid ITIM motif consists of the sequence (Ile/Val/Leu/Ser)-X-Tyr-X-X-(Leu/Val), where X denotes any amino acid (4). Ligand binding induces clustering of the inhibitory receptors and results in tyrosine phosphorylation that provides a docking site for the recruitment of cytoplasmic phosphatases that have Src-homology-2 (SH2) domains, including SHP-1 (SH2 domain-containing protein tyrosine phosphatase 1) and SHP-2. These phosphatases remove phosphate from tyrosine residues in the activated receptor and adaptors, such as SH2-binding leukocyte phosphoprotein of 76 kD (SLP-76), linker for activation of T cells (LAT), and CD3**ζ** (14) (Figure 1).

CTLA4 carries an ITIM-like YVKM motif, which associates with SHP-2 and reduces proximal TCR signaling through dephosphorylation of targets such as the TCR–CD3ζ complex, LAT, and ZAP-70 (15, 16), thereby inhibiting cell cycle progression and cytokine production. PD-1 has an ITIM motif as well as an immunoreceptor tyrosine-based switch motif (ITSM) (17). Both motifs appear to be phosphorylated following interaction with ligands, resulting in the recruitment of SHP-2 and possibly SHP-1; the co-localization of PD-1 with TCR microclusters induces dephosphorylation of CD3ζ, ZAP70, and PKC (17, 18). The TIGIT (T-cell immunoglobulin and ITIM domain) protein contains an ITIM motif and an immunoglobulin tail tyrosine (ITT)-like motif; phosphorylation of the tyrosine residue in either of these motifs is sufficient for signal transduction and inhibitory activity (19, 20). T-cell immunoglobulin-3 (TIM-3) does not have a classical signaling motif in its cytoplasmic tail (21). To simplify the descriptions in the present review, I am avoiding mention of several other remarkable signaling pathways that employ other phosphatases and intracellular motifs of the CTLA4, PD-1, TIM-3, and TIGIT co-inhibitory receptors; those topics have been covered in a number of excellent detailed reviews (3, 4, 11, 22).

**Figure 2:**
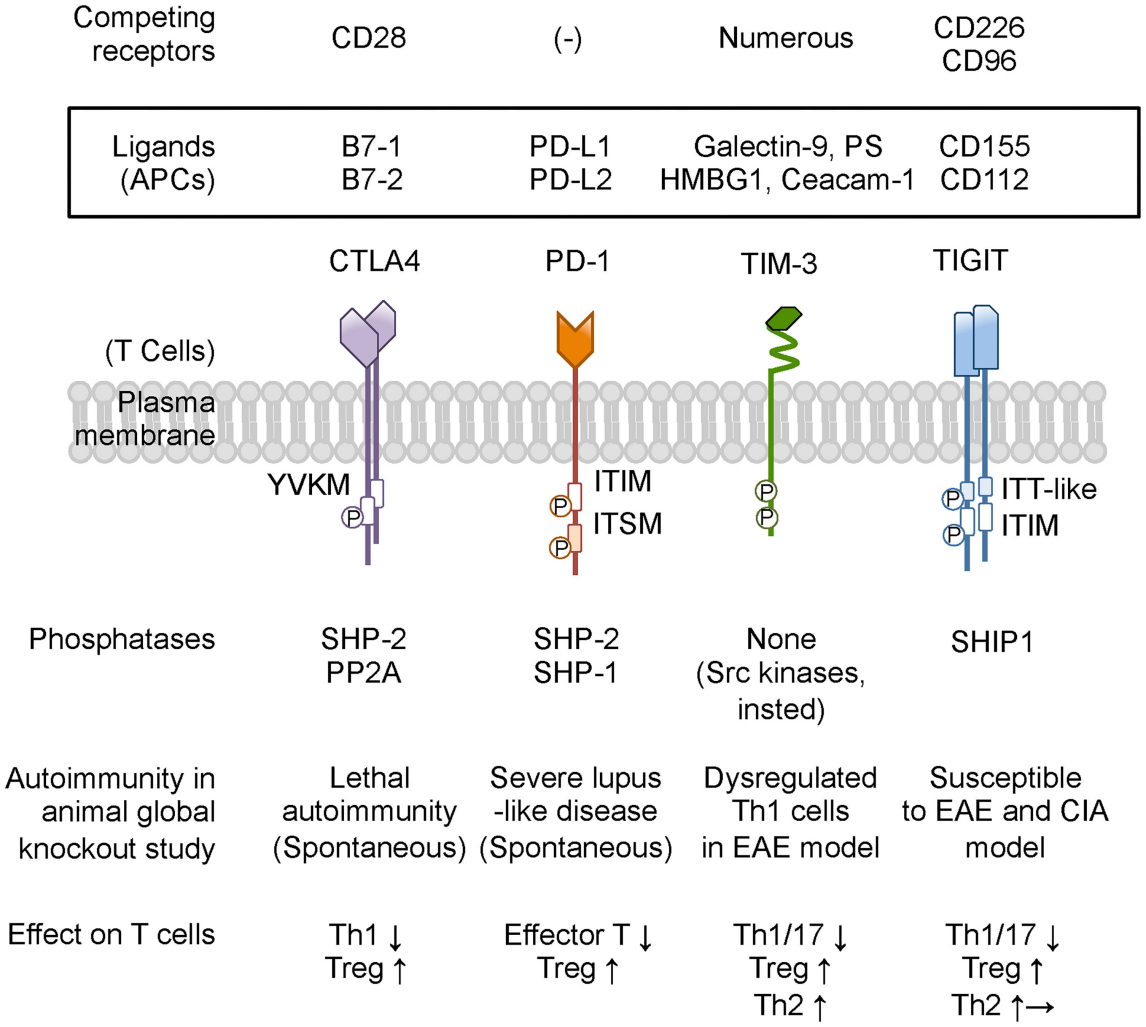
Co-inhibitory receptors on T cells and ligands, phosphatase, and modulation of T-cell function. Co-inhibitory receptors have a variety of physiological and pathological ligands, and the binding is occasionally competed by other immune receptors on T cells. TIM-3 does not have a classical signaling motif in its cytoplasmic tail. However, the cytoplasmic domain of TIM-3 contains tyrosine residues to be targets for phosphorylation and promoting TIM-3-mediated T-cell inhibition by allowing binding of SH2 domain-containing Src kinases. Based on animal studies, CTLA4 and PD-1 are predicted to be associated with more susceptibility to autoimmune disease, while neither TIM-3-deficient nor TIGIT-deficient mice present any spontaneous autoimmune disease phenotype. Co-inhibitory receptors on T cells commonly suppress effector T-cell functions by down-modulating Th1 cells and Th17 cells and by enhancing Treg cells. In addition, TIM-3 and TIGIT shift the cytokine balance to Th2 immunity. PS, phosphatidyl serine; HMGB1, high-mobility group protein B1; Ceacam-1, carcinoembryonic antigen-related cell adhesion molecule 1; PP2A, protein phosphatase 2A; EAE, experimental autoimmune encephalomyelitis; CIA, collagen-induced arthritis. Other abbreviations are shown in Figure 1 caption.

Collectively, co-inhibitory receptors regulate T-cell immunity by using both shared and unique signaling pathways (3), suggesting the specialized functions and providing the rationale for therapies that treat autoimmune glomerulonephritis by targeting these inhibitory receptors. The following subsection addresses the expression on T cells, function *in vivo*, and potential role for regulating autoimmune glomerular diseases of CTLA4, PD-1, TIM-3, and TIGIT, summarized in Figure 2 and Table 1.

**Table 1:** Experimental treatment models targeting T-cell co-inhibitory signaling in autoimmune diseases.

**Target receptor**	**Treatment**	**Animal model**	**Effect**	**References**
CTLA-4	CTLA4-Ig fusion	Spontaneous lupus	Suppress autoantibody production and prolong survival	(23)
		Spontaneous lupus GN (NZB/W F1)	When used in combination with cyclophosphamide, reduce renal inflammation and injury	(24)
		Spontaneous immune complex GN (Lyn^−/−^)	Not effective	(25)
		Anti-GBM (mouse/rat)	Controversial; depend on experimental conditions	(26–29)
PD-1	PD-L1-Ig fusion	Autoimmune GN	Reduce number of glomerular T cells and severity of glomerular damage	(30)
		T-cell-induced colitis	Suppress Th1 and Th17 response and ameliorate colitis	(31)
		CIA	Suppress T-cell response and ameliorate arthritis	(32, 33)
TIM-3	Galectin-9 (TIM-3 ligand)	Anti-GBM GN	Suppress T-cell response and ameliorate GN	(34)
		CIA	Suppress Th17 response and ameliorate arthritis	(35)
		EAE	Suppress Th1 response and ameliorate encephalomyelitis	(36)
TIGIT	TIGIT-Ig	Lupus GN	Reduced proteinuria and autoantibody, improve survival	(37)
	TIGIT-Ig and TIGIT tetramer	CIA	Suppress Th1 and Th17 response and ameliorate arthritis	(38)
	Agonistic antibody	EAE	Suppress Th1 and Th17 response and ameliorate encephalomyelitis	(39)

*CTLA4, cytotoxic T-lymphocyte-associated protein 4; Ig, immunoglobulin; NZB/W, New Zealand black/white; GN, glomerulonephritis; GBM, glomerular basement membrane; PD-1, programmed cell death 1; PD-L1, programmed cell death ligand 1; CIA, collagen-induced arthritis; TIM-3, T-cell immunoglobulin 3; EAE, experimental autoimmune encephalomyelitis; TIGIT, T-cell immunoglobulin and ITIM domain*.

### CTLA-4

#### Expression, Ligands, and General Function

CTLA4 is a potent negative regulator of T-cell response and was identified as a member of the CD28 family (40). Although CD28 is constitutively expressed on all naive CD4^+^ and CD8^+^ T cells and Treg cells, CTLA4 is transiently expressed on the surface of activated T cells (41, 42). The B7 family of proteins, B7-1 and B7-2, provides the major co-stimulatory signal for augmenting and sustaining T-cell responses through interaction with CD28 (42). B7-1 and B7-2 are shared ligands of CTLA4; the interaction of CTLA4 with these ligands leads to co-inhibitory signaling (43). In other words, the inhibitory mechanisms of CTLA4 include CD28 out-competition and blockade of intracellular signaling pathways, as CTLA4 has a 10-fold higher affinity than CD28 for B7-1 binding (43).

CTLA4-Ig (“abatacept”) is a biological that binds to B7-1 and B7-2, blocking both activating CD28-mediated signaling and inhibitory CTLA4-mediated signaling (44, 45) and effectively inhibiting naive antigen-specific CD4^+^ T-cell responses (46, 47). While the total CD4^+^ memory T-cell response was effectively attenuated by administration of CTLA4-Ig (48, 49), examinations of subsets of CD4^+^ helper T cells revealed that interleukin (IL)-17-secreting CCR6^+^ memory Th17 cells were resistant to CD28 and CTLA4 blockade (50).

#### Involvement in Autoimmunity and Glomerular Diseases and Therapeutic Model

CTLA4-deficient mice exhibit severe lymphoproliferative disease, with infiltration of activated T cells into various organs and death within a few weeks of birth (51–53). Given the promising results of CD28 and CTLA4 blockade in small animal models (23), strategies to target this pathway were developed in several clinical trials for the treatment of autoimmunity. CTLA4-Ig has been used clinically for the effective treatment of rheumatoid arthritis (RA) and juvenile idiopathic arthritis, as reviewed in (54), and has been tested against allergen-induced airway inflammation (55), ulcerative colitis (56), systemic lupus erythematosus (SLE) (57, 58), and other autoimmune diseases, as reviewed in (59). The results of those trials indicated that abatacept did not alter the inflammatory response to allergen challenge or show any efficacy in ameliorating colitis symptoms (59). Collectively, abatacept might be efficacious in the treatment of Th1-mediated autoimmune disease, such as RA, but remain less effective in the treatment of Th2- or Th17-mediated autoimmune disease such as asthma and inflammatory bowel disease (7). However, several studies have indicated that CTLA4-Ig protein attenuates glomerular injury in experimental models of crescentic glomerulonephritis (26–29). As a result, abatacept has been tested clinically in patients with granulomatosis with polyangiitis; the drug was well-tolerated in this population, providing a high frequency of disease remission and steroid discontinuation (60). A further clinical trial of abatacept in ANCA-associated vasculitis (AAV) has been progressed (https://clinicaltrials.gov/).

### PD-1

#### Expression, Ligands, and General Function

PD-1 originally was identified as an inducible surface receptor during programed cell death (61) and was shown to be expressed on stimulated T, B, and myeloid cells (62). PD-L1 and PD-L2 are two independent ligands for PD-1. PD-L2 expression is largely confined to dendritic cells (DCs) and monocytes/macrophages, but PD-L1 is more widely distributed on leukocytes and non-hematopoietic cells (63, 64). Expression of PD-L in peripheral tissue may regulate the behavior of infiltrating leukocytes (65). The PD-1/PD-L1 pathway exerts important inhibitory function in primary T-cell proliferation, cytokine production, cytotoxic activity, and cell survival (66, 67). This pathway also promotes development and function of Treg cells (68) and negatively regulates effector T-cell reactivation and function (69).

#### Involvement in Autoimmunity and Glomerular Diseases and Therapeutic Model

PD-1-deficient mice develop autoantibody-induced disease in a strain-dependent fashion; this autoimmune disease includes lupus-like glomerulonephritis leading to late death (70–72), although the phenotype in these mice is much milder than that of CTLA4-deficient mice. Consistent with human evidence of polymorphisms associated with SLE, T1D (Type 1 diabetes mellitus), and RA, experimental animal models of T1D-prone mice (73) and collagen-induced arthritis (CIA) (32) indicate that PD-1 activation attenuates autoimmune disease.

In the kidney, renal DCs have been shown to express PD-L1 and can be involved in suppressing CD4^+^ T-cell proliferation (74). Studies in experimental autoimmune glomerulonephritis have shown that blockade of PD-1/PD-1L interaction aggravates glomerular injury and cellular infiltration (64) and that activation of PD-1 using a PD-L1 fusion protein leads to a reduction in disease severity (30). Moreover, recent work with *PD-L1*^−/−^ mice showed that dosing with PD-L1 provided protection in a crescentic glomerulonephritis model via Treg-mediated suppression of the Th1 immune response (75). However, studies on immune-complex-mediated glomerulonephritis (induced by immunization with a foreign antigen) showed that blockade of the PD-1/PD-L axis (by antibody administration) did not reveal any significant pathological changes (76). This result suggests the need for careful interpretation of the roles of PD-1/PD-L in experimental autoimmune glomerulonephritis. Indeed, clinical trials testing the treatment of glomerulonephritis with PD-1 have not been reported. Thus, the clinical efficacy of modifying this pathway still requires further preclinical investigation.

### TIM-3

#### Expression, Ligands, and General Function

TIM-3 was identified as molecule expressed specifically in interferon (IFN)-γ-producing Th1 and CD8^+^ cytotoxic T cells, but not in naive T cells (77). Galectin-9 is a soluble S-type lectin that is widely expressed on immune and non-immune cells and has been shown to bind to the IgV domain of TIM-3, resulting in negative regulation of Th1 immunity (36). In addition to galectin-9, phosphatidyl serine, high-mobility group protein B1 (HMGB1), and carcinoembryonic antigen-related cell adhesion molecule 1 (Ceacam-1) have been identified as TIM-3 ligands (78–80). It remains to be determined whether the triggering of TIM-3 by individual ligands or by combinations thereof has distinct impacts on TIM-3 function.

#### Involvement in Autoimmunity and Glomerular Diseases and Therapeutic Model

TIM-3 can be protective in autoimmunity but often is sparsely expressed; in contrast, the protein is highly expressed in cancer and chronic viral infection, resulting in the dampening of protective immunity (22). Even so, multiple reports have shown that TIM-3 blockade results in abrogation of peripheral tolerance of Th1-cell-mediated responses. Anti-TIM-3 blocking murine model develops hyper-acute experimental autoimmune encephalomyelitis (EAE) (77); treatment with soluble TIM-3-Ig results in T-cell hyper-activation and IFN-γ production (81). In addition to its role in regulating effector T-cell responses, TIM-3 also may have a role in regulating the function of Foxp3^+^ Treg cells (82). Several studies have shown that TIM-3^+^ Treg cells have superior suppressive function when compared to TIM-3^−^ Treg cells (82, 83).

Galectin-9 is a rare example of agonistic treatment based on a natural ligand. Administration of galectin-9 as a soluble protein in mouse ameliorates EAE and CIA (35, 36). The indiscriminate nature of galectin avidity, such that the molecule binds to sugars on multiple different glycoproteins, makes it difficult to definitively attribute these effects to TIM-3 signaling rather than to the manipulation of another galectin-9 binding partner (84). Nevertheless, administration of galectin-9 ameliorates experimental anti-GBM glomerulonephritis; this protective role is associated with inhibition of Th1 and Th17-cell-mediated immune responses and enhanced Th2 immunity in the kidney (34).

Human studies suggest that renal TIM-3 and galectin-9 expression levels are higher in immune-complex-mediated glomerulonephritis, such as IgA nephropathy (85) and lupus nephritis (86) compared to the control group. Some investigations have examined the expression of TIM-3 on peripheral blood cells and in the serum of patients with glomerular diseases (87, 88), but there is little evidence of a role for TIM-3 in other types of autoimmune glomerulonephritis. Clinical trials evaluating the treatment of autoimmune glomerulonephritis using an agent targeting TIM-3 have not been reported. Although the targeting of TIM-3 signaling holds potential for the treatment of T-cell-mediated glomerulonephritis, further preclinical investigation will be required to elucidate effects both on different immune cells and on ligand binding partners other than galectin-9.

### TIGIT

#### Expression, Ligands, and General Function

TIGIT was discovered as a novel member of the CD28 protein family (89, 90). TIGIT is expressed on activated T cells, memory T cells, a subset of Treg cells, and follicular helper T (Tfh) cells, and binds to two ligands, CD155 and CD112, that are expressed on APCs (19, 89, 90). CD226 and CD96 bind to the same ligands, and the CD226–CD155 interaction mediates a co-stimulatory response in cytotoxic T cells (89). TIGIT competes with CD226 by binding with greater affinity to CD155–CD112 to disrupt that co-stimulatory effect, thereby resulting in a dominant inhibitory effect (22). In this regard, the pathway formed by CD226, TIGIT, and their ligands resembles the B7-CD28/CTLA4 pathway: in both cases, a pair of receptors—one positive, one negative–share ligands expressed on APCs (22).

#### Involvement in Autoimmunity and Glomerular Diseases and Therapeutic Model

In human, genomic analyses showed that a polymorphism in CD226 (Gly307Ser) is linked to multiple autoimmune diseases, including T1D, multiple sclerosis, and RA (91, 92). Although TIGIT-deficient mice do not develop spontaneous autoimmunity, these animals display augmented T-cell responses upon immunization (93). The function of TIGIT was examined in EAE and CIA models, with results suggesting that TIGIT is protective for the pro-inflammatory Th1 and Th17 cellular response and contributes to peripheral tolerance (38, 93). In addition to its direct inhibitory role in effector T cells, TIGIT also inhibits immune responses by promoting Treg function and IL-10 production (22). Curiously, several lines of evidence indicates that TIGIT signaling can shift the cytokine balance away from a Th1- and Th17-cell-dominated response and toward a Th2-cell-like response, concurrently with TIGIT-binding-mediated CD155 signaling on APCs (89, 94).

Given the similarity between the B7-CD28/CTLA4 and CD155/CD112-TIGIT/CD226 signaling pathways with regard to their co-signaling frameworks, TIGIT-Ig theoretically should block both activating CD226 signaling and inhibitory TIGIT signaling in a manner similar to that of CTLA-4-Ig. In addition, TIGIT-Ig induces CD155 signaling in cultured DCs *in vitro* and decreases IL-10 production by Th1 cells *in vivo* (89). The specific difference between these two pathways is that B7 is expressed primarily in professional APCs, while CD155 is expressed by a variety of non-professional APCs such as the vascular endothelium, fibroblasts, and tumor cells (95). When autoimmune disease occurs, the tissue that is infiltrated by T cells contains mainly non-professional APCs, and the CD155/CD112-TIGIT/CD226 pathway might be involved in tissue damage. Still, in both human and animal models, few studies have examined the role of TIGIT signaling in renal-specific disease. Although the treatment of a murine lupus model (NZB/NZW F1 mice) using TIGIT-Ig significantly improved survival, inflammatory responses, and glomerular damage (37), preclinical studies on other glomerular diseases will be needed to permit clinical use of TIGIT-Ig.

## The Development of Autoimmune Glomerulonephritis Caused by Immune Checkpoint Inhibitors

In the past decade, cancer therapy has been revolutionized by the development of drugs that promote immune-mediated tumor destruction (96). CTLA-4 and PD-1/PD-L1 are the two best-studied co-inhibitory pathways (97); the use of antibodies as immune checkpoint inhibitors, anti-CTLA4 antibodies, and anti-PD-1/PD-L1 antibodies has been approved for the treatment of several cancers (98–100). While these immunotherapies have shown striking success, blockade of CTLA-4 and PD-1/PD-L1 are associated with adverse effects that resemble autoimmune disorders, including SLE, RA, thyroiditis, and T1D (59, 101). Additionally, renal vasculitis, immune-complex-mediated glomerulonephritis, and pauci-immune glomerulonephritis recently have been reported (102–108). Most systemic vasculitis cases resolved with either holding the immune checkpoint inhibitors and/or administering glucocorticoids (109). These evidences imply relationship between interventional blocking co-inhibitory receptor signaling and development of renal vasculitis, suggesting that this pathway may be a therapeutic target.

## Rationale for Targeting Th1/17 Effector and Regulatory T Cells in Autoimmune Vasculitis

As mentioned before, blockade of inhibitory receptors occasionally has resulted in renal vasculitis as well as lupus-like autoimmunity. While autoantibodies play a role in a number of forms of glomerulonephritis, renal vasculitis in humans features the infiltration of T cells and macrophages (110, 111), suggesting a delayed hypersensitivity reaction in kidney. Given that autoreactive CD4^+^ and CD8^+^ cells are present in vasculitis patients (112–115), experimental passive transfer studies have defined a role for CD4^+^ and CD8^+^ cells in AAV (116, 117). CD4^+^ effector T cells, particularly upon differentiation to Th17 cells, mediate production of neutrophil chemoattractants by tissue cells via release of IL-17A and renal injury (118, 119). Studies using mice deficient in Th1- and Th17-defining cytokines have shown an initial Th17-dominant lesion followed later by a Th1-dominant outcome (120). Moreover, as human studies implicate abnormal CD4^+^ Foxp3^+^ Treg number and function in AAV patients (121–124), depletion of Treg cells led to more anti-neutrophil cytoplasmic protein-specific T cells and more severe glomerulonephritis (125). Approaches for targeting inhibitory receptors might (in theory) include inhibitory receptor-Ig fusion proteins, ligand-Ig fusion proteins, artificial ligands, and agonistic antibodies, as well as the use of bi-specific antibodies to co-ligate inhibitory and activating receptors (59). Among these approaches, as shown in Table 1, TIGIT-Ig protein, agonistic anti-TIGIT antibodies, and TIM-3 ligands (e.g., galectin-9), along with PD-L1-Ig and CTLA4-Ig proteins, should be considered candidates for development as bench-to-bedside therapeutics for treatment of T-cell-mediated autoimmune glomerulonephritis through regulation of the function of Th1/Th17 and Treg cells.

## Conclusion

The studies in knockout mice and clinical experiences of vasculitis caused by immune checkpoint inhibitors treatment give numerous indications that the loss of a functional co-inhibitory receptor leads to sensitivity for autoimmune disease. Clinical utilization of co-inhibitory axes has not progressed in autoimmune disease as it has in cancer. Except for CTLA4-Ig, no clinical trial on co-inhibitory targeted therapy for autoimmune vasculitis and lupus nephritis has been progressed until the middle of 2020. Nevertheless, some recent studies have shown preclinical evidence for the utility of targeting co-inhibitory receptors in lupus and glomerulonephritis. As effector T cells and Treg function have a pivotal role in the development of autoimmune vasculitis, currently available CTLA4-Ig has been tested to evaluate the efficacy of achieving glucocorticoid-free remission in patients with relapsing vasculitis. Clinical efficacy of therapy targeting at PD-1, TIM-3, and TIGIT is expected to be not always consistent because their ligands are different from each other and each co-inhibitory receptor utilizes unique cell signaling as well as shared pathway. Therefore, clinical trials on modulating co-inhibitory signaling by Ig-fusion protein, agonistic antibody, and natural ligands should be carefully designed after sufficient preclinical investigations with clinically relevant animal models show that effector T cells are precisely involved in antigen-specific disease development. Toward the development of effective therapies for T-cell-mediated autoimmune glomerulonephritis, it would be preferable to pay attention to the impact and features of these inhibitory pathways in immunological renal disease settings.

## Author Contributions

The author confirms being the sole contributor of this work and has approved it for publication.

## Conflict of Interest

The author declares that the research was conducted in the absence of any commercial or financial relationships that could be construed as a potential conflict of interest.
